# SenSeqNet: A Deep Learning Framework for Cellular Senescence Detection From Protein Sequences

**DOI:** 10.1111/acel.70344

**Published:** 2025-12-23

**Authors:** Hanli Jiang, Dongliang Deng, Yu Yuan, Jianyu Ren, Xin Yang, Siyi Liu, Bin Tan, Li Lin, Lubin Liu

**Affiliations:** ^1^ Department of Molecular Genetics University of Toronto Toronto Ontario Canada; ^2^ Lunenfeld‐Tanenbaum Research Institute, Mount Sinai Hospital Sinai Health Toronto Ontario Canada; ^3^ Computational Biology Program Ontario Institute for Cancer Research Toronto Ontario Canada; ^4^ Department of Computer Science University of Toronto Toronto Ontario Canada; ^5^ Department of Oncology Chongqing Traditional Chinese Medicine Hospital Chongqing China; ^6^ Department of Obstetrics and Gynecology Women and Children's Hospital of Chongqing Medical University Chongqing China; ^7^ Department of Obstetrics and Gynecology Chongqing Health Center for Women and Children Chongqing China; ^8^ Department of Computer Science University of Wisconsin – Madison Madison Wisconsin USA; ^9^ Department of Electrical and Computer Engineering University of Illinois Urbana‐Champaign Urbana Illinois USA; ^10^ Department of Obstetrics and Gynecology The First Affiliated Hospital of Chongqing Medical University Chongqing China

**Keywords:** cellular senescence, deep learning, protein language model

## Abstract

Cellular senescence, defined as the irreversible arrest of cell proliferation in response to stress, contributes to tissue dysfunction and drives the progression of age‐related diseases. Accurate detection of senescent states is therefore essential for understanding aging mechanisms and identifying therapeutic targets. However, conventional laboratory assays are time‐consuming and difficult to scale. Here, we present SenSeqNet, a deep learning framework that predicts cellular senescence directly from protein sequences. SenSeqNet integrates embeddings from the Evolutionary Scale Modeling (ESM‐2) with a hybrid LSTM–CNN architecture to capture both sequential and higher‐order structural features. The model achieved 86.43% accuracy in independent testing, outperforming traditional machine learning and deep learning approaches. Importantly, the high‐confidence genes predicted by SenSeqNet were significantly enriched in canonical senescence‐associated pathways, indicating that the model captures biologically coherent regulatory programs rather than overfitting to sequence labels. These results establish SenSeqNet as a robust and biologically informed tool for senescence detection and provide a foundation for accelerating research into aging and age‐related therapeutics.

## Graphical abstract

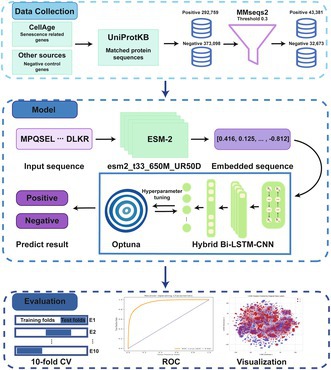

## 1 Introduction

Aging is a complex biological process marked by the gradual accumulation of cellular and molecular damage over time, leading to diminished physiological function and the onset of age‐related diseases (Flatt 2012). The incidence rates of diabetes, heart disease, neurological disorders, and various cancers increase with age (Partridge et al. 2018). Therefore, the early prediction and detection of cellular senescence play a vital role in preventing the development of numerous diseases (Childs et al. 2015). Carlos López‐Otín et al. have identified loss of proteostasis as a key hallmark of aging (López‐Otín et al. 2023). Recent advances in high‐throughput sequencing and proteomics have generated an unprecedented volume of biological data, presenting new opportunities to explore aging at the molecular level (Aebersold and Mann 2016; Gorgoulis et al. 2019). Protein sequences are of particular interest, as they encode the structural and functional properties of proteins that mediate nearly all cellular processes (Bepler and Berger 2021). Analyzing these sequences to identify signs of aging could reveal key biomarkers and therapeutic targets, paving the way for novel interventions in age‐related conditions (Moaddel et al. 2021).

Traditional methods for studying protein sequences have often relied on manual analysis and rule‐based approaches, which are limited in their capacity to uncover the complex patterns associated with aging (Chen et al. 2024). However, machine learning (ML) with the emergence of deep learning (DL) revolutionized how biological data is analyzed, enabling the automatic extraction of complex patterns and insights that are difficult to obtain with traditional methods (Lin et al. 2024; Ashiqur Rahman et al. 2021). In particular, DL models, with their ability to automatically learn hierarchical representations from raw data, have demonstrated remarkable success in various biomedical applications, including protein structure prediction, drug discovery, and disease diagnosis (Tran et al. 2021; Zhou et al. 2022; Crampon et al. 2022).

**FIGURE 1:**
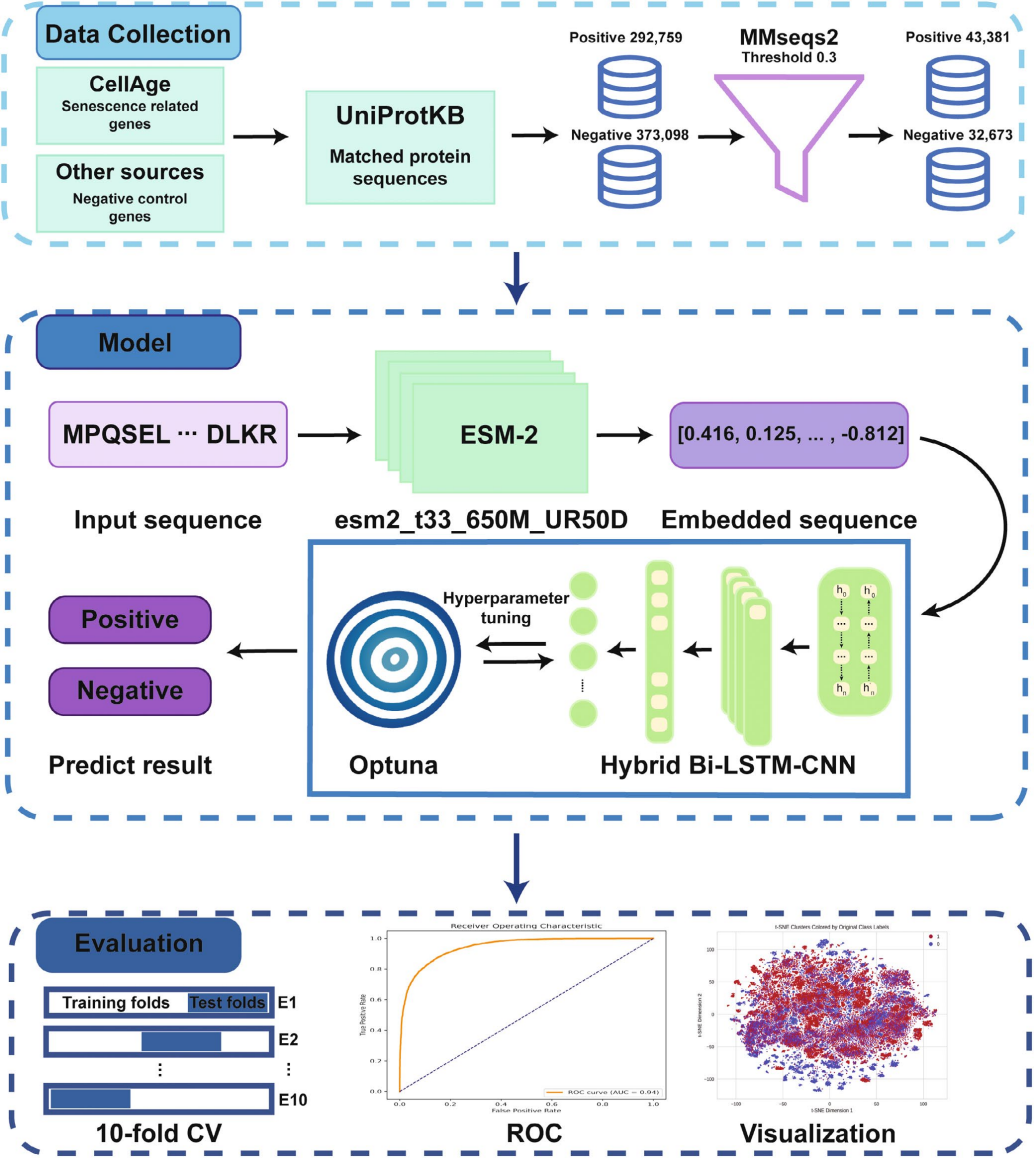
The overall framework.

Despite significant advancements in DL, the application of these techniques to detect cell aging using protein sequences remains relatively scarce. Recently, the integration of multiple DL models to leverage their respective advantages has demonstrated significant success (Jiao et al. 2024). To further explore the application of integrated DL models, we developed SenSeqNet (Figure 1), a novel DL approach that begins with ESM2 for feature extraction and then synergistically combines the strengths of long short‐term memory (LSTM) networks and convolutional neural networks (CNNs) (Dastider et al. 2021). LSTM networks are adept at modeling sequential data like protein sequences, capturing long‐term dependencies that are critical for understanding the temporal aspects of aging (Tsukiyama et al. 2021). Meanwhile, CNNs excel at extracting spatial features (Du et al. 2023), making them ideal for identifying complex patterns within the outputs generated by LSTM networks.

The next crucial step in sequence‐based protein prediction tasks is choosing the optimal model architecture, with the representation of sequence data being a key factor in determining overall model performance (Cui et al. 2021). We utilized the Evolutionary Scale Modeling (ESM) (Lin et al. 2023), a state‐of‐the‐art transformer‐based model renowned for its ability to capture deep evolutionary relationships in protein sequences. Specifically, we evaluated several variants of the ESM‐2 model to generate embeddings for our task. To gain insights into the high‐dimensional data structure, we conducted visualization analysis using the t‐distributed Stochastic Neighbor Embedding (t‐SNE) algorithm (Van der Maaten and Hinton 2008). This allowed us to observe distinct clustering patterns within the protein sequence embeddings, highlighting the effectiveness of our embedding approach. While other protein language models (PLMs) such as ProtTrans (Elnaggar et al. 2022), ESM‐1b (Rao et al. 2020), ESM‐1v (Meier et al. 2021) and ProteinBERT (Brandes et al. 2022) were considered, their sequence length limitations (e.g., 1024 amino acids) made them less suitable for our dataset, which contains nearly 6000 protein sequences exceeding this limit. ESM‐2, with its support for sequences exceeding 1024 amino acids, was chosen for its ability to handle longer sequences while preserving global context, resulting in consistent and robust performance in preliminary experiments. We also compared the t‐SNE visualizations between ESM‐2 and other traditional feature extraction methods. ESM‐2 outperforms other approaches in producing more clearly defined and separable clusters. Through extensive experimentation, we evaluated multiple high performance ML and DL models to identify the optimal architecture for classifying cell aging. We compared these models based on their ability to capture unique patterns in protein sequences related to cellular senescence. Among these, SenSeqNet achieved the best performance in both cross‐validation and independent external testing, reflecting its ability to jointly model sequential and higher‐order spatial features. Importantly, SenSeqNet also enabled gene‐level interpretation: genes predicted with the highest confidence were significantly enriched in canonical senescence‐associated pathways. This concordance between predictive accuracy and biological pathway relevance indicates that SenSeqNet not only discriminates senescence‐related sequences but also converges on mechanistic signaling programs that underpin cellular aging.

## 2 Materials and Methods

### 2.1 Positive Gene Set Construction

Senescence‐associated genes were first curated from the CellAge database. The database can be accessed at https://genomics.senescence.info/cells/index.html. To ensure functional specificity, only genes that were experimentally validated to induce cellular senescence in vitro (i.e., annotated as “Senescence Effect = induce”) were retained, while genes labeled as unclear or with no reported senescence‐inducing effect were excluded. This process yielded 163 high‐confidence senescence‐inducing genes, as listed in Table S1.

To capture senescence states observed in physiological contexts, we further incorporated three in vivo senescence resources: SASP Atlas, SenMayo genes (Saul et al. 2022), and SenPy (Sanborn et al. 2025). Genes consistently identified in at least two of these datasets were retained, yielding 47 additional in vivo‐supported senescence signature genes. These genes represent conserved and tissue‐relevant senescence states across diverse biological systems. The full gene list and source annotations are provided in Table S2. In total, 210 senescence‐associated genes were included in the positive set.

### 2.2 Negative Gene Set Construction

To construct the negative gene set, we selected genes associated with cellular states that are functionally distinct from senescence. These included genes involved in cytoskeletal maintenance, housekeeping and ribosomal functions, cell cycle and proliferative signaling, programmed cell death (apoptosis), core metabolic processes, and receptor‐mediated signaling. These pathways collectively represent non‐senescent physiological or proliferative cellular conditions, providing a biologically meaningful contrast to senescence. In total, 148 non‐senescent genes were included in the negative set. The full list of negative genes and their functional annotations is provided in Table S3.

### 2.3 Protein Sequence Retrieval, Redundancy Filtering, and Dataset Splitting

Protein sequences corresponding to all positive and negative gene sets were retrieved from the Universal Protein Knowledgebase (the UniProtKB database) (UniProt Consortium 2021). Because the predictive model operates on the protein sequence level, all annotated protein isoforms for each gene were collected. To reduce redundancy and avoid model bias caused by overrepresented sequence families, the initial dataset of 292,759 positive and 373,098 negative protein sequences was clustered using MMSeqs2 (Steinegger and Söding 2017) with an identity threshold of 0.3. This clustering procedure yielded a final representative dataset of 43,381 positive and 32,673 negative sequences. Only these representative sequences were used for subsequent model training and validation to avoid data leakage (Bernett et al. 2024). The dataset was divided into a training set (80%) and a validation set (20%) for model development and performance evaluation. The code and benchmark dataset are publicly available at https://github.com/HanliJiang13/SenSeqNet, ensuring transparency and reproducibility of the study.

### 2.4 External Validation of SenSeqNet

To assess the generalizability of SenSeqNet, we performed external validation using two in vitro datasets (GSE13330 and GSE63577) and one in vivo dataset (SenCID; Tao et al. 2024). GSE13330 and GSE63577 profile human fibroblasts undergoing replicative or DNA damage–induced senescence, while SenCID provides experimentally verified senescence signatures from human tissues. In total, 26 senescence‐related genes were collected from these datasets, and their corresponding protein sequences were processed using the same UniProtKB retrieval. The validated gene names and their dataset sources are provided in Table S4.

### 2.5 SenSeqNet Model Structure

**FIGURE 2:**
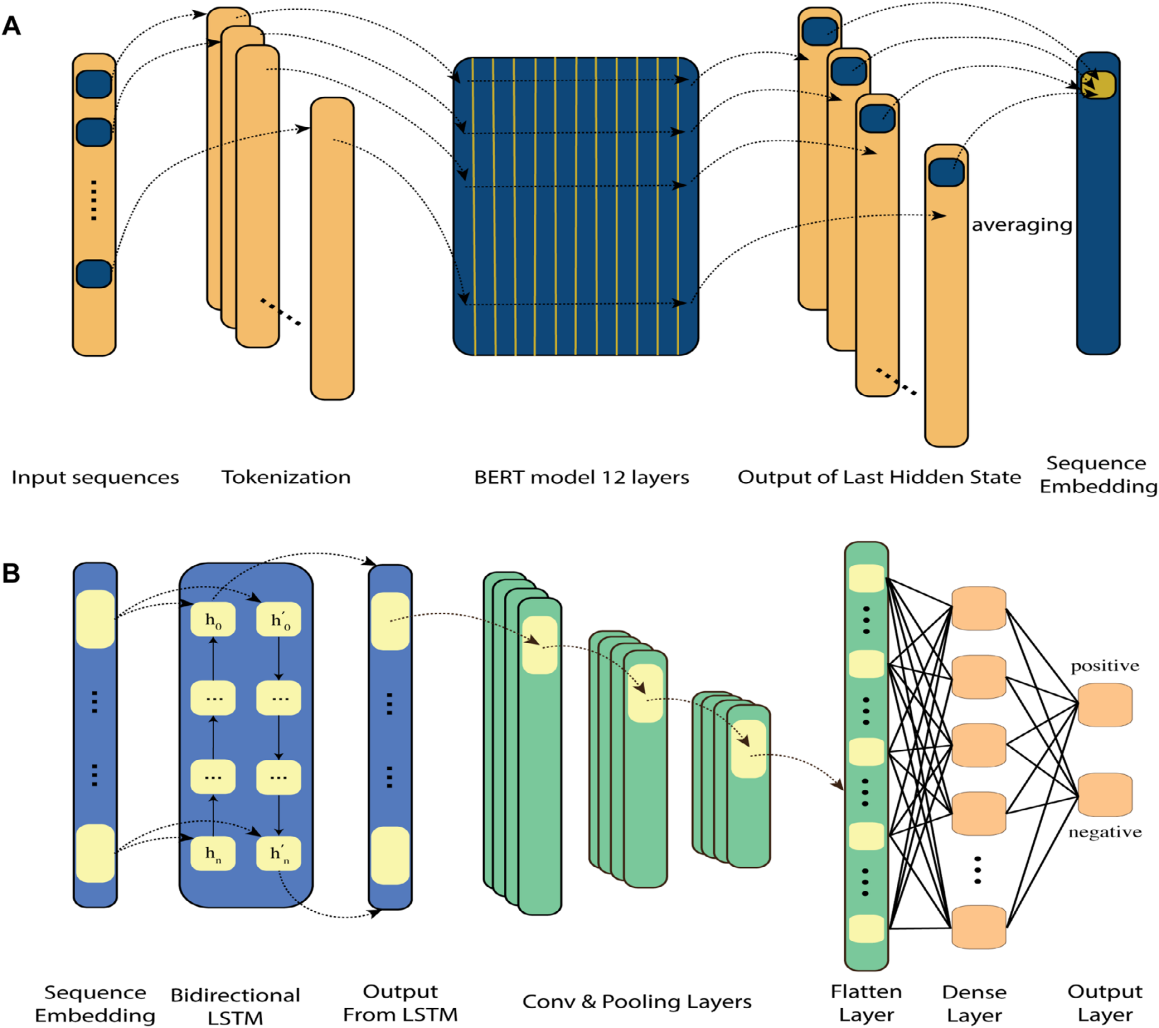
Sample Architecture of the SenSeqNet: (A) Evolutionary Scale Modeling (ESM‐2) architecture for feature extraction. (B) Hybrid long short‐term memory–Convolutional Neural Network (LSTM–CNN) model.

As illustrated in Figure 2, SenSeqNet consists of two main components: ESM‐2 and the hybrid LSTM‐CNN architecture. Beginning with the first component, effective representation of sequence data is an essential step in building a robust prediction model, especially when dealing with protein sequences (LeCun et al. 2015). Thus, we employed the ESM‐2 (Lin et al. 2023), known for its exceptional ability to capture deep evolutionary relationships in protein sequences (Figure 2A). ESM‐2 is trained on approximately 65 million unique protein sequences using a masked language modeling objective, which involves predicting the identity of randomly selected amino acids within a sequence based on their surrounding context (Du et al. 2023). This training enables ESM‐2 to learn the dependencies between amino acids, allowing it to effectively capture both local and global contextual information.

The unsupervised nature of the ESM‐2 model's training allows it to internalize sequence patterns across diverse proteins, effectively capturing structural information linked to these patterns (Nguyen and Hy 2024). ESM‐2 is highly effective at processing and embedding from large‐scale protein datasets, making it particularly suitable for sequence representation tasks. The model's performance across various bioinformatics applications has been well‐documented (Mall et al. 2024), demonstrating its ability to discern subtle patterns within sequences, which are important for accurate prediction tasks. There are multiple ESM‐2 models, each differing in architectural configuration, where an increase in the number of transformer layers corresponds to a proportional increase in the number of model parameters. To identify the most effective variant for our dataset, we evaluated each variant's output and utilized the results to train two distinct models: a CNN to assess the spatial features and an LSTM to analyze the sequential dependencies within the results. By balancing the accuracy, computational efficiency, and scalability of these models, we determined the most effective ESM‐2 variant for our dataset. The selected model variant was then used for embedding generation throughout our study. The detailed results of this evaluation will be discussed in the Results section.

Followed by ESM‐2, the embedded sequences are input into the second main component, a hybrid LSTM‐CNN architecture (Figure 2B). In this setup, a Bidirectional LSTM network is first employed to process the sequence data, leveraging its capacity to model temporal dependencies in both directions, thus capturing comprehensive long‐term relationships between amino acids in the protein sequences. These sequential features are then passed to the CNN, which focuses on identifying spatial patterns within the LSTM‐extracted features. This combined approach enables the model to capture both temporal and spatial characteristics. This two‐part architecture, combining the evolutionary context captured by ESM‐2 with the temporal and spatial analysis capabilities of the LSTM‐CNN, enables SenSeqNet to effectively identify crucial features in protein sequences. This comprehensive approach significantly enhances the model’s ability to distinguish senescence‐related proteins from non‐senescent proteins, as demonstrated by the results.

### 2.6 Comparative Models for Benchmarking SenSeqNet

In this study, we explored several ML and DL architectures to develop a reliable model for detecting cell aging using protein sequences (LeCun et al. 2015). Given the lack of prior research in this specific domain of cell aging, our approach involved comprehensive experimentation with a range of models commonly employed in protein sequence prediction to understand their capabilities and limitations. The architectures evaluated include CNNs (Zhang et al. 2023), Recurrent Neural Networks (RNNs) (Müller et al. 2018), LSTMs (Tsukiyama et al. 2021), and bidirectional LSTMs. Recognizing the strengths of both CNNs in extracting spatial features and bidirectional LSTMs in capturing sequential dependencies (Khademi et al. 2022), we also experimented with hybrid models that combined these two architectures. The combined CNN and bidirectional LSTM model was designed to leverage the advantages of both, aiming to improve the accuracy and robustness of cell aging detection from protein sequences. Additionally, we constructed several ML models, including Bagging (Breiman 1996), Support Vector Machines (SVM) (Hearst et al. 1998), Random Forest (RF) (Breiman 2001), and XGBoost (Chen and Guestrin 2016), to provide a comparative understanding of their performance against DL approaches. Below, we present a comprehensive description of each DL model. Hyperparameters such as learning rate, dropout rate, batch size, number of layers, and hidden dimension were optimized for each model using Optuna (Akiba et al. 2019), a framework for hyperparameter optimization. To illustrate parameter dependence, we provide a representative sensitivity analysis for the LSTM variant of SenSeqNet (Figure S1), showing how variation in these hyperparameters influences model performance across the Optuna search.

#### 2.6.1 Convolutional Neural Network

This study introduces a custom CNN Classifier for sequence prediction, employing a multilayer CNN architecture. The model consists of three convolutional layers, each with a varying number of filters (79, 111, and 180 filters, respectively) to extract spatial features from the input sequences. Max‐pooling layers follow the first two convolutional layers to reduce the spatial dimensions of the feature maps, and batch normalization is applied after each convolutional layer to stabilize training. The flattened feature maps are then passed into fully connected layers consisting of 512 and 256 neurons to learn higher‐level feature representations. Dropout is applied between the fully connected layers to prevent overfitting, and the final output layer produces multi‐class predictions using a log softmax activation function. This model efficiently captures both local and global feature representations through the combination of convolutional and fully connected layers.

#### 2.6.2 Recurrent Neural Networks

This study introduces a custom RNN Classifier for sequence prediction, utilizing a multilayer RNN architecture. The model incorporates an RNN with multiple layers to capture temporal dependencies in the input sequences. The hidden layers of the RNN consist of a specified number of units, optimized for extracting sequential features. A batch normalization layer is applied to the output of the last time step to stabilize training and improve convergence. The model's sequential output is further passed through fully connected layers consisting of 512 and 256 neurons, respectively, to capture higher‐level feature representations. Dropout is applied between the fully connected layers to reduce overfitting. This model efficiently captures both temporal patterns and higher‐order representations through its recurrent and fully connected layers.

#### 2.6.3 Long Short‐Term Memory Network

The LSTM Classifier we used incorporates LSTM layers that are designed to capture long‐term dependencies in the input sequences by considering both past and future information at each time step. Specifically, the model first processes input sequences through a stacked LSTM layer containing 427 LSTM units, allowing it to retain relevant information across time steps. The LSTM is set with multiple layers (num_layers = 5) to improve its capacity to capture complex temporal relationships. The output from the final LSTM layer, representing the last time step, is passed through a dropout layer to mitigate overfitting. This is followed by a fully connected layer, which transforms the LSTM output into multi‐class predictions. The model effectively leverages the sequential nature of LSTMs.

#### 2.6.4 Bidirectional LSTM

The Bi‐LSTM Classifier extends the capabilities of traditional LSTMs by incorporating bidirectional LSTM layers. These layers are uniquely designed to process information both forwards and backwards along the input sequence, enabling the model to capture context from both the past and future at each time step. This dual‐direction processing allows for a more comprehensive understanding of temporal relationships within the data. The Bi‐LSTM architecture features a bidirectional LSTM layer with 250 hidden units distributed across four layers, ensuring that each time step is enriched with full sequence context. Such an arrangement not only enhances the model's ability to discern patterns that a single‐direction LSTM might miss but also improves predictive accuracy by integrating insights from the entire sequence breadth.

#### 2.6.5 Combined Architectures

CNNs are particularly adept at capturing spatial information, making them highly effective for tasks that involve the analysis of grid‐like data structures, such as images or sequences with local spatial dependencies. Additionally, LSTMs excel in handling sequential data, allowing for the modeling of long‐term dependencies and temporal patterns, which are crucial in many predictive tasks. The integration of these two models aims to create a model that synergizes the spatial feature extraction capabilities of CNNs with the sequence modeling strengths of LSTMs. To explore the potential of this combination, we investigated several architectural strategies:

##### 2.6.5.1 CNN Followed by LSTM (CNN‐LSTM)

This architecture begins with the CNN, which extracts spatial features from the raw protein sequences. The CNN is adept at identifying local patterns or motifs within the sequence data that may be associated with cell aging. These spatial features are then fed into the Bidirectional LSTM, which captures the sequential dependencies among the CNN‐extracted features. The Bidirectional LSTM's ability to analyze the sequence data from both directions ensures that critical temporal information is not overlooked.

##### 2.6.5.2 Parallel LSTM and CNN (pLSTM‐CNN)

In this approach, the Bidirectional LSTM and CNN operate simultaneously on the input data. The Bidirectional LSTM processes the sequence data to capture temporal dependencies from both past and future directions, while the CNN extracts spatial features independently. The outputs of both models are then combined to form a comprehensive representation of the protein sequences, which is used for the final classification. This parallel architecture is designed to maximize the strengths of both models, capturing a wide range of information from the data by considering both spatial and sequential aspects simultaneously.

### 2.7 Evaluation Metrics

The following commonly used evaluation metrics were employed to assess the effectiveness of the models (Ao et al. 2022): sensitivity (Sn), specificity (Sp), Matthews correlation coefficient (MCC) (Chicco and Jurman 2020), accuracy (Acc), F1 score, and AUC. The formulas for these metrics are provided below:

$$\mathrm{Sn}=\frac{\mathrm{TP}}{\mathrm{TP}+\mathrm{FN}}$$

$$\mathrm{Sp}=\frac{\mathrm{TN}}{\mathrm{TN}+\mathrm{FP}}$$

$$\mathrm{MCC}=\frac{\left(\mathrm{TP}\times \mathrm{TN}\right)-\left(\mathrm{FP}\times \mathrm{FN}\right)}{\sqrt{\left(\mathrm{TP}+\mathrm{FP}\right)\left(\mathrm{TP}+\mathrm{FN}\right)\left(\mathrm{TN}+\mathrm{FP}\right)\left(\mathrm{TN}+\mathrm{FN}\right)}}$$

$$\mathrm{Acc}=\frac{\mathrm{TP}+\mathrm{TN}}{\mathrm{TP}+\mathrm{FP}+\mathrm{FN}+\mathrm{TN}}$$

$$F1\;\text{score}=\frac{2\times \mathrm{TP}}{2\times \mathrm{TP}+\mathrm{FP}+\mathrm{FN}}$$

where: True positive (TP): The number of correctly identified cases of cell aging; True negative (TN): The number of correctly identified non‐aging cases; False positive (FP): The number of non‐aging cases incorrectly identified as aging; False Negative (FN): The number of aging cases incorrectly identified as non‐aging; AUC represents the area under the receiver operating characteristic (ROC) curve and is used to evaluate the overall performance of the model across different thresholds.

### 2.8 Gene‐Level Performance Assessment and Pathway Analysis

We evaluated our model's performance at the gene level and conducted pathway analysis. The trained model generated predictions for all protein sequences, classifying each as senescence‐associated or not. To determine gene‐level performance, we aggregated predictions across all protein sequences belonging to each gene, calculating the proportion of correctly classified sequences as the per‐gene accuracy score. This approach accounted for genes with multiple protein products and provided a robust measure of classification performance. To identify a high‐confidence set of senescence‐associated genes, we applied stringent filtering criteria: (1) genes must be supported by at least 30 test sequences to ensure statistical reliability, and (2) genes must achieve a classification accuracy of at least 0.9. These thresholds were selected to balance between maintaining high confidence and retaining sufficient genes for downstream analysis. The resulting high‐confidence gene set was subjected to pathway enrichment analysis using g:Profiler web server (Reimand et al. 2007, 2016). Enrichment significance was assessed using the hypergeometric test with false discovery rate (FDR) correction for multiple testing. Pathways with FDR < 0.05 were considered significantly enriched. The gene ratio for each pathway was calculated as the proportion of high‐confidence genes present in the pathway relative to the total pathway size.

## 3 Results and Discussion

### 3.1 ESM Model Selection

As detailed in the Methods section, we evaluated several variants of the Evolutionary Scale Modeling (ESM) for feature extraction from our protein sequence dataset. The goal was to identify the model that would provide the most accurate features while maintaining computational efficiency. Here, we present the results of this evaluation and the rationale for selecting the optimal ESM variant. The ESM variants evaluated included ESM‐2_t6, ESM‐2_t12, ESM‐2_t30, and ESM‐2_t33, each varying in complexity and embedding dimensions. The performance of these models was assessed based on their accuracy from our LSTM‐CNN architecture (Table 1).

**TABLE 1:** Comparison of ESM‐2 model variants for feature extraction.

Model	#Layers	#Params (M)	#Emb Dim	Acc (%)
ESM‐2_t6	6	8	320	79.15
ESM‐2_t12	12	35	480	82.26
ESM‐2_t30	30	150	640	84.16
ESM‐2_t33	33	650	1280	86.43

**FIGURE 3:**
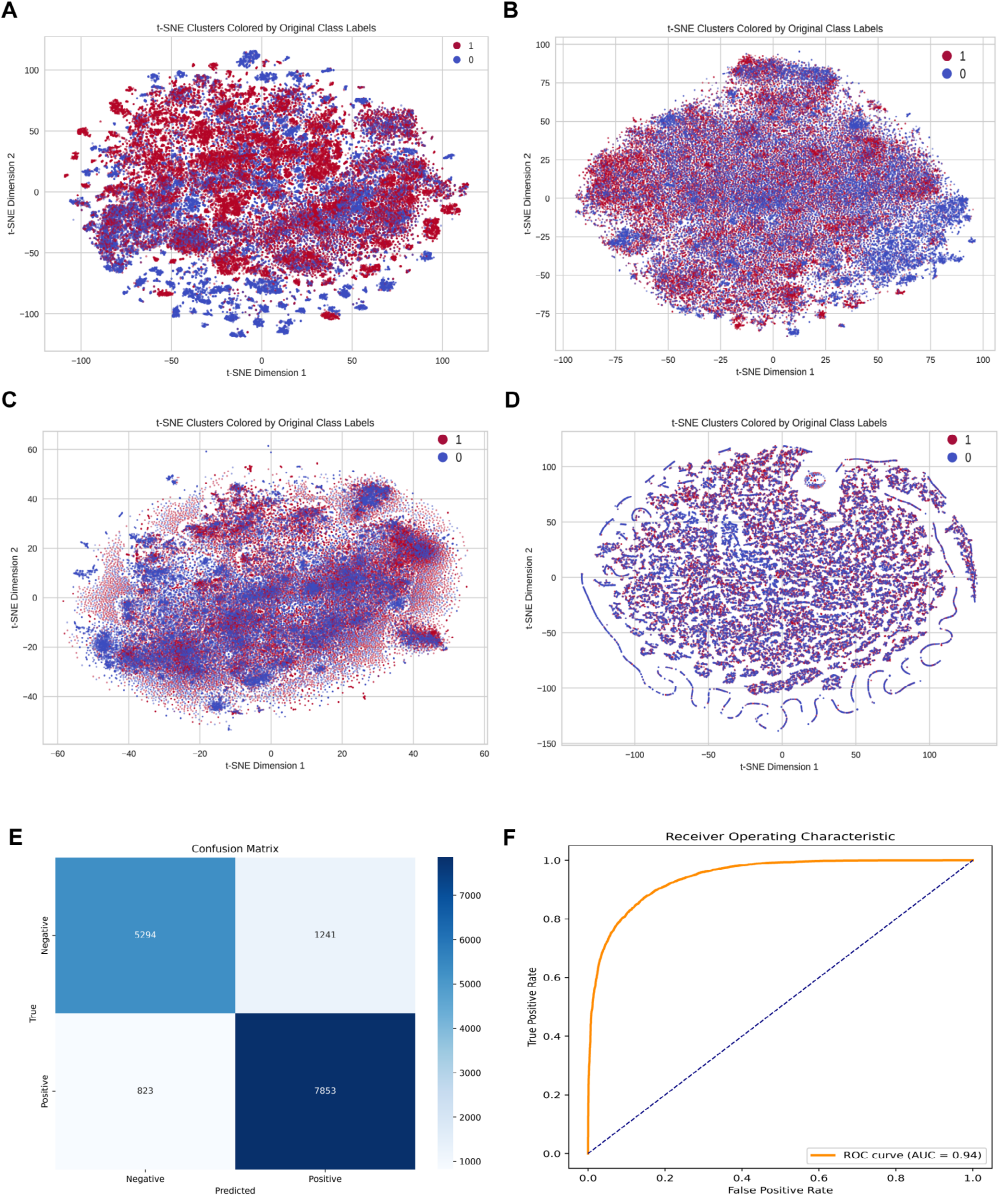
t‐SNE visualization for ESM‐2 and traditional methods (A–D): (A) t‐SNE visualization for the ESM‐2 model. (B) t‐SNE visualization for the ACC. (C) t‐SNE visualization for the CKSAAP. (D) t‐SNE visualization for the GTPC. (E) Confusion matrix for the SenSeqNet, illustrating a balanced classification outcome. (F) Receiver Operating Characteristic (ROC) curves for the SenSeqNet models. The curves highlight the superior sensitivity and specificity of SenSeqNet.

The ESM‐2_t33 model achieved the highest accuracy at 86.43%, outperforming the other variants. This accuracy advantage makes ESM‐2_t33 the best choice for feature extraction. Therefore, we have selected ESM‐2_t33 for use throughout our experiments. To provide a more intuitive illustration of how ESM‐2_t33 embeddings differentiate the classes, we performed t‐SNE on the extracted features (Figure 3A). In contrast to the manual feature extraction methods—ACC (Figure 3B), CKSAAP (Figure 3C), and DDE (Figure 3D)—which exhibit heavily overlapping clusters, the ESM‐2_t33 embeddings form distinct groupings that closely align with the original class labels. This clear separation indicates that ESM‐2_t33 captures critical sequence‐level signals pertinent to senescence, rather than arbitrary noise. Moreover, the t‐SNE plots offer a complementary perspective to numerical metrics such as accuracy, specificity, and MCC, thereby reinforcing our confidence that ESM‐2_t33 provides a robust foundation for downstream classification tasks.

### 3.2 Comparative Performance of Multiple Protein Language Models

To assess the generalizability of SenSeqNet, we extracted features from multiple PLMs, including ProtTrans (T5), ProteinBERT, ESM‐1b, ESM‐1v, and ESM‐2, and subsequently fed these embeddings into our LSTM‐CNN architecture. Table 2 presents the performance metrics across all tested configurations.

**TABLE 2:** Comparative performance of using embeddings from ProtTrans, ProteinBERT, ESM‐1b, and ESM‐2.

PLM	Acc%	Sn%	Sp%	F1‐score%	MCC
ProtTrans (T5)	51.54	31.70	68.71	37.77	0.0044
ProteinBERT	60.94	41.83	77.48	49.84	0.2073
ESM‐1b	84.57	83.86	**85.47**	85.92	0.6898
ESM‐1v	83.48	84.53	82.27	84.52	0.6680
ESM‐2	**86.43**	**90.51**	81.01	**88.38**	**0.7221**

*Note:* Bold values represent the highest value achieved for each metric among all compared models.

Our results reveal a stark contrast between the ESM series and the other PLMs evaluated. ESM‐2 achieved the highest accuracy (86.43%), with strong sensitivity (90.51%), specificity (81.01%), and an F1‐score of 88.38%. ESM‐1b and ESM‐1v also exhibited robust performance, attaining accuracies of 84.57% and 83.48%, respectively. These findings suggest that ESM‐based embeddings capture key senescence‐related patterns in protein sequences, even when considering longer sequences exceeding 1024 residues.

ESM‐2 was selected for several key reasons. First, scalability and efficiency: ESM‐2 processes longer sequences (over 1024 residues) without requiring extensive chunking and merging, but feature extraction with ESM‐1b and ESM‐1v required approximately twice as much computational time compared to ESM‐2. Second, active development: As the most recent iteration of the ESM family, ESM‐2 benefits from ongoing updates, improving scalability and offering potential for further accuracy gains. Third, comparable high‐accuracy: ESM‐2 achieved slightly higher accuracy (86.43% vs. 84.57%), superior sensitivity (90.51%), and a robust Matthews correlation coefficient (MCC) of 0.7221, ensuring reliable predictions.

In contrast, ProtTrans (T5) and ProteinBERT, even when applying standard chunking and averaging strategies to handle sequences exceeding 1024 residues, performed significantly worse than ESM‐based models. ProtTrans (T5) achieved only 51.54% accuracy, 31.70% sensitivity, 68.71% specificity, an F1‐score of 37.77%, and an MCC of 0.0044, reflecting near‐random classification. ProteinBERT performed moderately better, with 60.94% accuracy, but still fell far short of the performance achieved by ESM‐based embeddings.

These results underscore the challenges faced by more generalized PLMs in distinguishing senescence‐associated proteins from non‐senescent proteins. In contrast, ESM‐2 provided robust performance, computational efficiency, and scalability, making it the optimal choice for SenSeqNet and well‐suited for large‐scale analyses of aging‐related proteins.

### 3.3 Comparative Performance Evaluation of SenSeqNet With Other Deep Learning Models

We compared the performance of SenSeqNet with several DL models, including CNN, RNN, LSTM, Bidirectional LSTMs, and various hybrid models. Each model was tested to determine its effectiveness in detecting cell aging from protein sequences, focusing on key performance metrics such as accuracy, AUC, Sn, Sp, and MCC using an independent test set. The comparative results of these models are summarized in Table 3.

**TABLE 3:** The performance comparison of SenSeqNet with other deep learning models on the independent test set.

Model	Acc%	Sn%	Sp%	F1‐score%	MCC
CNN	80.38	82.33	77.78	82.72	0.6002
RNN	80.66	82.92	77.66	83.02	0.6055
LSTM	81.30	85.68	75.47	83.94	0.6165
BiLSTM	81.89	81.73	82.10	83.73	0.6343
CNN‐LSTM	78.02	72.85	82.47	74.16	0.5570
pLSTM‐CNN	80.94	78.30	**83.22**	79.23	0.6164
**SenSeqNet**	**86.43**	**90.51**	81.01	**88.38**	**0.7221**

*Note:* Bold values represent the highest value achieved for each metric among all compared models.

The CNN, known for its ability to capture spatial features, achieved an accuracy of 80.38% and an MCC of 0.6002. While effective in identifying local patterns within the protein sequences, the CNN struggled to capture temporal dependencies critical for detecting cellular senescence. To address this limitation, RNNs were introduced to model sequential data. The RNN showed a small improvement, with an accuracy of 80.66% and an MCC of 0.6055, indicating its capability to better capture sequential patterns in the data. Next, more advanced temporal models, such as LSTMs, were explored. The LSTM, designed to handle long‐term dependencies in sequential data, further improved accuracy to 81.30%, with an MCC of 0.6165. Building on this, the Bidirectional LSTM, which processes information in both forward and backward directions, enhanced the ability to capture complex dependencies, achieving an accuracy of 81.89% and an MCC of 0.6343. This model excelled at processing temporal patterns but lacked focus on spatial feature extraction. We also evaluated several hybrid architectures. Among these, the CNN‐LSTM configuration demonstrated the lowest performance, with an accuracy of 78.02% and an MCC of 0.5570, suggesting that processing spatial features before sequential features may not fully leverage the strengths of both architectures. The pLSTM‐CNN model performed better, achieving an accuracy of 80.94% and an MCC of 0.6164, though it had higher computational demands due to the simultaneous processing of spatial and sequential features.

The best‐performing architecture was the SenSeqNet, which effectively combined the LSTM's ability to capture sequence dependencies with the CNN's proficiency in refining spatial features. SenSeqNet achieved the highest accuracy of 86.43%, an AUC of 0.94, and an MCC of 0.7221. These results demonstrate that the combination of LSTM and CNN architectures, when sequenced in this order, leads to a more comprehensive understanding of protein sequences, resulting in superior predictive accuracy. Overall, the results highlight that while single models such as CNNs and LSTMs are effective in their respective domains of spatial or temporal feature extraction, the hybrid approach of SenSeqNet provides the best of both worlds, outperforming both individual models and other hybrid configurations. The confusion matrix and ROC curve (Figure 3E,F) further confirm the stability and generalizability of SenSeqNet in detecting cellular senescence. To better understand the model's misclassifications, we conducted detailed gene‐level analysis (Table S5), which revealed that false positives predominantly occurred in specific gene families including ribosomal proteins, while true senescence markers showed consistently low error rates, providing biological insight into the model's decision boundaries.

### 3.4 Comparative Analysis of SenSeqNet With Machine Learning Models

To further evaluate the performance of SenSeqNet, we also compared its performance with various commonly used ML models including RF, XGBoost, SVM, and Bagging. The highest accuracy achieved among these machine models was 79.67% by RF, while XGBoost showed a notably high Sn of 94.19% but compromised Sp at 57.95%. Overall, none of the traditional models surpassed 80% in accuracy, with moderate MCC ranging between 0.5521 and 0.5844. The results are summarized in Table 4.

**TABLE 4:** Comparative analysis of SenSeqNet with machine learning models on the independent test set.

Model	Acc%	Sn%	Sp%	F1‐score%	MCC
LR	77.75	77.39	78.24	79.87	0.5521
RF	79.67	89.91	66.06	83.46	0.5844
XGBoost	78.62	**94.19**	57.95	83.40	0.5735
SVM	78.90	77.48	80.82	80.90	0.5770
Bagging	78.80	84.75	70.09	82.01	0.5644
**SenSeqNet**	**86.43**	90.51	**81.01**	**88.38**	**0.7221**

*Note:* Bold values represent the highest value achieved for each metric among all compared models.

In comparison, SenSeqNet exhibited consistently superior performance, achieving the highest accuracy, specificity, F1‐score, and MCC, highlighting its effectiveness in extracting complex, nonlinear features from large‐scale protein sequence data. This disparity in performance underscores the capability of SenSeqNet to better handle the extensive and intricate relationships inherent in biological datasets, especially when dealing with large volumes of data, where traditional ML models may struggle to fully capture contextual dependencies.

### 3.5 External Validation on Independent Senescence‐Associated Genes

To further validate SenSeqNet's predictive capability and simulate real‐world usage scenarios, we tested the model on an external dataset comprising 26 senescence‐related genes (Table S4) that were not included in any phase of model training or development. This dataset yielded 52,227 protein sequences for validation. SenSeqNet classified 40,502 of these 52,227 sequences as senescence‐associated, achieving a true positive rate of 77.55% (Figure S2). Detailed per‐sequence and per‐gene validation results are provided in Tables S6 and S7, respectively, which include both binary predictions (positive/negative) and their associated confidence scores. The strong performance on these independent sequences confirms SenSeqNet's ability to generalize beyond its training data. This validation approach directly mirrors real‐world applications where researchers encounter newly discovered or poorly characterized proteins that require classification without prior experimental validation, demonstrating SenSeqNet's utility as a practical tool for identifying senescence‐associated sequences in exploratory research contexts.

### 3.6 Per‐Gene Performance and Top Enriched Pathways

**FIGURE 4:**
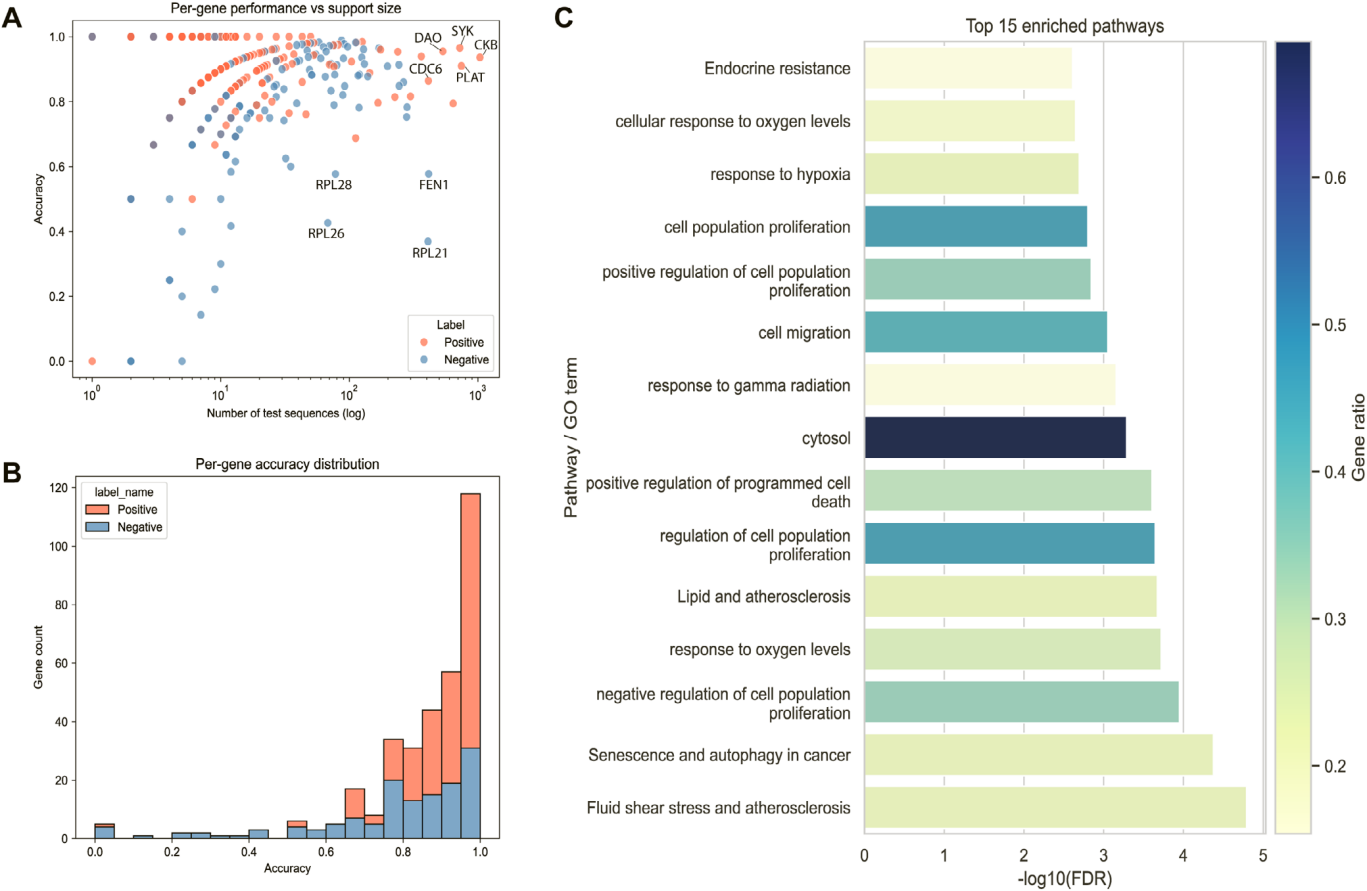
Per‐gene performance and top enriched pathways (A–C): (A) Scatter plot of per‐gene accuracy versus the number of test sequences (log scale), with points colored by class label (positive vs. negative). Highlights which genes have reliable high‐accuracy because they're supported by many sequences versus those that are high but data‐poor. (B) Stacked histogram showing the distribution of per‐gene accuracy for positive and negative labels. Makes it easy to see that most positives cluster above ~0.9 while negatives span a broader range, especially when support is limited. (C) Horizontal bar chart of the 15 most significant enriched pathways from the high‐confidence gene set. Bar length encodes −log_10_(FDR) (higher = more significant), while bar color reflects the gene ratio (overlap/total high‐accuracy genes).

To evaluate the model's ability to identify senescence‐associated genes, we assessed per‐gene classification performance across our test set (Figure 4). The relationship between per‐gene accuracy and the number of supporting test sequences revealed that senescence‐related genes (positive class) consistently achieved high‐accuracy scores, with most clustering above 0.9 accuracy when supported by sufficient sequence data (Figure 4A). Notably, the model demonstrated exceptional performance on well‐validated senescence markers such as *CKB*, *SYK*, and *DAO*, all of which achieved near‐perfect accuracy despite having large numbers of test sequences. In contrast, certain ribosomal protein genes including *RPL21*, *RPL26*, and *RPL28* showed markedly poor performance despite adequate sequence support, suggesting these genes may possess sequence features that confound the model's senescence‐specific patterns. This pattern was further confirmed by the distribution analysis, which showed that positive genes predominantly concentrated in the high‐accuracy range (> 0.9), while negative control genes exhibited a broader accuracy distribution, particularly when sequence support was limited (Figure 4B). To identify robust biological signals, we selected high‐confidence positive genes meeting stringent criteria (> 30 supporting sequences and > 0.9 accuracy), which yielded a set of reliably classified senescence‐associated genes. Pathway enrichment analysis of these high‐confidence genes revealed strong enrichment for established senescence‐related pathways, with the most significant associations found in fluid shear stress and atherosclerosis, senescence and autophagy in cancer, and negative regulation of cell population proliferation (Figure 4C). Notably, pathways related to positive regulation of cell population proliferation, cell migration, and response to oxygen levels all showed significant enrichment, consistent with known senescence biology where these processes are dysregulated.

## 4 Conclusion

In this study, we demonstrate the efficacy of a novel hybrid deep learning model, SenSeqNet, which integrates ESM‐2 embeddings with a hybrid LSTM‐CNN architecture to detect senescence‐related proteins from protein sequences. Our approach successfully leverages the sequential modeling capabilities of LSTMs and the spatial feature extraction strengths of CNNs to capture both temporal and spatial patterns within protein sequences that are indicative of cellular senescence. The SenSeqNet proved to be the optimal model, achieving an impressive accuracy of 86.43% and an AUC of 0.94 on the independent test set. The SenSeqNet outperformed both single models and other hybrid configurations, underscoring the importance of sequence processing order in DL architectures. To further evaluate SenSeqNet's reliability, we performed 10‐fold cross‐validation, achieving a final cross‐validated accuracy of 86.13% ± 0.53%, and validated the model on an external senescence dataset, where it achieved an accuracy of 77.55%. These results confirm the robustness and generalizability of SenSeqNet.

Gene‐level analysis revealed that SenSeqNet exhibits remarkable specificity for senescence‐associated proteins, achieving near‐perfect accuracy for well‐established senescence markers such as *CKB*, *SYK*, and *DAO*. SenSeqNet successfully distinguishes senescence‐specific signatures from general housekeeping or stress‐response proteins. This discriminatory capability demonstrates that SenSeqNet has learned biologically meaningful patterns rather than merely detecting general cellular dysfunction. Pathway enrichment analysis of high‐confidence predictions further validated our approach, revealing significant enrichment in established senescence‐related pathways including fluid shear stress and atherosclerosis, senescence and autophagy in cancer, and negative regulation of cell population proliferation. These pathway associations align with known senescence biology.

Our findings highlight the potential of DL in advancing aging research by enabling accurate detection of aging at the cellular level. The robustness and scalability of the SenSeqNet model make it a valuable tool for large‐scale screening in biological sequencing, providing rapid initial predictions of senescence‐associated proteins. The model's ability to identify biologically coherent gene sets and pathways suggests its utility for hypothesis generation in aging research. For instance, the model could be employed to identify aging‐related proteins within the mitochondrial proteome, thereby offering valuable insights into mitochondrial dysfunction during aging. Furthermore, SenSeqNet could guide research into aging‐associated diseases, such as Alzheimer's disease and cardiovascular conditions, by pinpointing senescence‐related proteins that may serve as potential biomarkers or therapeutic targets. Additionally, the model could contribute to drug discovery efforts by identifying key aging‐related proteins for experimental validation and potential therapeutic intervention, particularly through the use of mouse models to test the efficacy of potential treatments. While it shows potential for identifying candidates for further study, its primary application lies in offering preliminary predictions and supporting subsequent validation efforts, rather than directly uncovering molecular mechanisms. This approach can significantly aid fundamental research by narrowing down targets for experimental investigation, ultimately contributing to a deeper understanding of cellular senescence.

However, a key limitation of this study is the model's interpretability, particularly in identifying biologically significant motifs within protein sequences. Unlike extensively studied proteins, such as antibodies, where known motifs and experimental validation are available, proteins associated with cellular senescence lack such data. While our pathway analysis provides biological validation at the systems level, understanding the specific sequence features driving individual predictions remains challenging. This gap limits our ability to confirm whether the model focuses on biologically meaningful patterns at the sequence level. Future research should address this challenge by exploring and validating potential motifs related to cellular senescence, potentially leveraging our high‐confidence gene predictions as a starting point for motif discovery. Enhanced interpretability would not only validate the model's biological relevance but also potentially reveal novel senescence‐associated sequence signatures, advancing both our understanding of cellular aging and the clinical applicability of the model's predictions.

## Author Contributions

Hanli Jiang and Dongliang Deng were responsible for the conceptualization and methodology development of the study, as well as drafting the original manuscript. Yu Yuan and Siyi Liu contributed to data curation and investigation. Jianyu Ren and Xin Yang were involved in model development and visualization. Lubin Liu, Bin Tan, and Li Lin provided supervision, acquired funding, and led the review and editing of the manuscript. All authors have reviewed and approved the final version of the manuscript.

## Funding

The work was supported by General Program of the National Natural Science Foundation of China (No. 82171622) and the National Natural Science Foundation of China for Youth (No. 82001573).

## Ethics Statement

The authors have nothing to report.

## Consent

The authors have nothing to report.

## Conflicts of Interest

The authors declare no conflicts of interest.

## Supporting information


**Table S1:** Curated CellAge senescence‐inducing genes (*n* = 163).

**Table S2:** Cross‐dataset in vivo senescence signature genes (*n* = 47).

**Table S3:** Functionally defined negative control gene set representing non‐senescent cellular states.

**Table S4:** External validation gene set and corresponding data sources.

**Table S5:** The full list of genes level performance in the study.

**Table S6:** Per‐sequence performance of the external validation.

**Table S7:** Per‐gene performance of the external validation.

**Figure S1:** Hyperparameter sensitivity of SenSeqNet‐LSTM. (A) Validation accuracy vs. LSTM hidden dimension. (B) Validation accuracy vs. dropout rate. (C) Validation accuracy vs. learning rate (log scale). (D) Distribution of validation accuracy by batch size.
**Figure S2:** Histogram of SenSeqNet's predicted positive probabilities on the external validation set (52,227 sequences). Bars show the distribution of predicted positive probability, with the red dashed line marking the 0.5 decision threshold.

## Data Availability

The data that support the findings of this study are available in SenSeqNet at https://github.com/HanliJiang13/SenSeqNet. These data were derived from the following resources available in the public domain: The Universal Protein Knowledgebase, https://www.uniprot.org/—The Database of Cell Senescence Genes, https://genomics.senescence.info/cells/—SenCID, https://doi.org/10.1016/j.cmet.2024.03.009—Senepy, https://doi.org/10.1038/s41467‐025‐57047‐7—Senmayo genes, https://doi.org/10.1038/s41467‐022‐32552‐1.
