# Supplementary material for: SenSeqNet: A Deep Learning Framework for Cellular Senescence Detection From Protein Sequences
Source: Aging Cell. 2025 Dec 23;25(1):e70344. doi: 10.1111/acel.70344 (PMC12724000; doi:10.1111/acel.70344)
Supplement: Supplementary file 8 — Figure S1: Hyperparameter sensitivity of SenSeqNet‐LSTM. (A) Validation accuracy vs. LSTM hidden dimension. (B) Validation accuracy vs. dropout rate. (C) Validation accuracy vs. learning rate (log scale). (D) Distribution of validation accuracy by batch size. Figure S2: Histogram of SenSeqNet's predicted positive probabilities on the external validation set (52,227 sequences). Bars show the distribution of predicted positive probability, with the red dashed line marking the 0.5 decision threshold. [file ACEL-25-e70344-s006.docx]

**S1 Figure.** Hyperparameter sensitivity of SenSeqNet-LSTM. A. Validation accuracy vs. LSTM hidden dimension. B. Validation accuracy vs. dropout rate. C. Validation accuracy vs. learning rate (log scale). D. Distribution of validation accuracy by batch size.


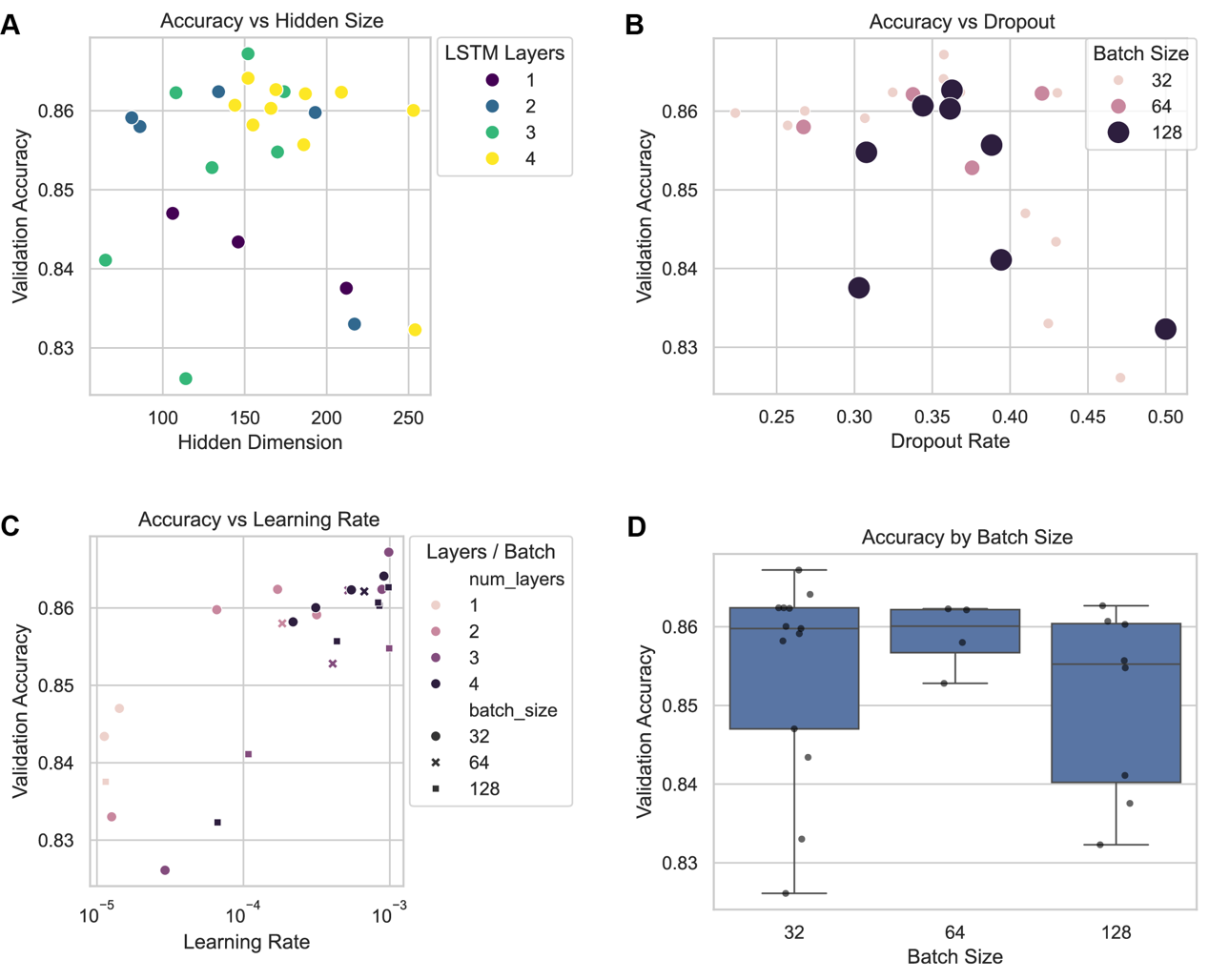


**S2 Figure.** Histogram of SenSeqNet’s predicted positive probabilities on the external-validation set (52,227 sequences). Bars show the distribution of predicted positive probability, with the red dashed line marking the 0.5 decision threshold.

**
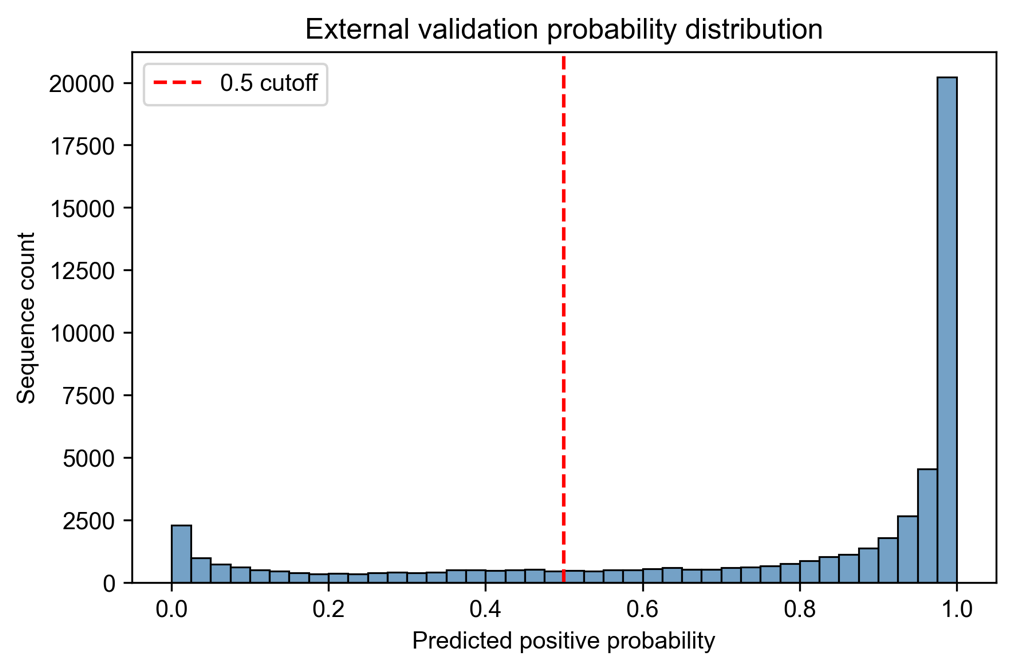
**
